# Impact of Pulmonary Vascular Resistances in Heart Transplantation for Congenital Heart Disease

**DOI:** 10.2174/157340311797484213

**Published:** 2011-05

**Authors:** Avihu Z Gazit, Charles E Canter

**Affiliations:** 1 Divisions of Critical Care and Cardiology, Department of Pediatrics, Saint Louis Children’s Hospital, Washington University in Saint Louis, Missouri, USA; 2 Division of Cardiology, Department of Pediatrics, Saint Louis Children’s Hospital, Washington University in Saint Louis, Missouri, USA

**Keywords:** Congenital heart disease, pulmonary vascular resistance, heart transplantation.

## Abstract

Congenital heart disease is one of the major diagnoses in pediatric heart transplantation recipients of all age groups. Assessment of pulmonary vascular resistance in these patients prior to transplantation is crucial to determine their candidacy, however, it is frequently inaccurate because of their abnormal anatomy and physiology. This problem places them at significant risk for pulmonary hypertension and right ventricular failure post transplantation. The pathophysiology of pulmonary vascular disease in children with congenital heart disease depends on their pulmonary blood flow patterns, systemic ventricle function, as well as semilunar valves and atrioventricular valves structure and function. In our review we analyze the pathophysiology of pulmonary vascular disease in children with congenital heart disease and end-stage heart failure, and outline the state of the art pre-transplantation medical and surgical management to achieve reverse remodeling of the pulmonary vasculature by using pulmonary vasodilators and mechanical circulatory support.

## INTRUDCTION

Heart transplantation (HT) is increasingly considered a treatment option for patients with congenital heart disease (CHD) and end-stage heart failure. According to the registry of the International Society of Heart and Lung Transplantation, 63% of infant heart recipients, and 25% of heart recipients aged 11 to 17 years between January 1996 and June 2009, carry the underlying diagnosis of CHD [1]. A recent analysis of the Pediatric heart Transplant Study registry [2] identified 488 children (6 months-18 years of age) with CHD at the time of listing for HT transplanted at 35 centers from January 1990 through December 2002. Patients<6 months of age were excluded from analysis due to differing listing algorithms in United States. The major diagnostic categories for the 488 study patients were single ventricle (36%), d-transposition of the great arteries (12%), right ventricular outflow tract lesions (most commonly Tetralogy of Fallot) (10%), ventricular/atrial septal defect (8%), l-transposition of the great arteries (8%), and complete atrioventricular canal defect (8%). 454 of patients had at least 1 operation before HT. Staged palliation for single ventricle, including the Norwood procedure, or variants of the Glenn procedure, was the last operation in approximately 20% of the patients, and the Fontan operation was the last surgical procedure in 107 patients, representing 22% of the CHD population. 

Congenital diagnosis remains a highly significant risk factor for mortality one and five years after HT [1]. Accurate listing of patients for HT requires assessment of the pulmonary vascular resistance (PVR) to avoid the potential of donor right heart failure [3, 4] Unfortunately, despite advances in perioperative management and careful preselection, pulmonary hypertension related right ventricular failure still occurs in pediatric HT recipients [5].

In patients with CHD, given the high post HT mortality risk, accurate assessment of PVR has increased importance, but paradoxically, these patients are often harder to assess. Anomalies within the pulmonary vasculature or dual supplies of pulmonary blood flow may make accurate calculations of resistance impossible. The sluggish pulmonary and hepatic blood flow in the Fontan circuit increases the risk for microemboli and arteriovenous malformations that may change the distribution of blood flow to the right and left lung. The assumption that elevated PVR may contribute to late Fontan failure even in patients with “normal” pre-HT PVR was studied by Mitchell and colleagues [6]. This group conducted a single center retrospective study of patients who underwent HT for failing Fontan and Kawashima circulations and compared pre- and post- HT cardiac catheterization pulmonary hemodynamics. They found post HT elevation of transpulmonary gradient (TPG) and PVR in all patients with late Fontan circulation failure (range 10-16 mmHg and 2.8-5.4 Wood units • m^2^, respectively) and concluded that “the increase in TPG and PVR to pathologic levels post HT reflects a fixed element of PVR that was unmasked with the introduction of normal pulmonary blood flow”. 

The risk of post HT right ventricular failure and mortality in children with heart disease and elevated PVR is well established and underlines the need for pre-transplantation assessment of TPG and PVR. The same experience in adults led to recommendations that HT should not be performed if the PVR index exceeds 6 Wood units • m^2 ^or if the TPG is greater than 15 mmHg. However, these recommendations do not take into account possible reversibility of elevated PVR. In fact, partial or complete reversibility of elevated PVR and TPG were observed acutely in response to short acting systemic and pulmonary vasodilators such as nitroprusside [7], prostaglandin E_1_ [8], and inhaled nitric oxide [9, 10], and in response to prolonged administration of a phosphodiesterase-5 inhibitor such as sildenafil [11-13], endothelin antagonist such as bosentan [14], and inotropic agents such as milrinone [15] dopamine and dobutamine [16]. Based on available reports, children who demonstrate PVR reversibility can successfully undergo orthotopic HT even if their PVR index exceeds 6 Wood units • m^2^ or if the TPG exceeds 15 mmHg (see discussion below: Hemodynamic unloading and regression of fixed pulmonary vascular resistance).

## PATHOPHYSIOLOGY AND NATURAL HISTORY

### (1) Effects of shear stress on the vascular endothelium

As blood flows through a vessel it exerts a physical force on the vessel wall. This force generates stress that can be resolved into two principal vectors. [1] The stress parallel to the vessel wall is defined as shear stress. This represents the frictional force that blood flow exerts on the endothelial surface of the vessel wall. [2] The stress perpendicular to the vessel wall is defined as tensile stress. This represents the dilating force of blood pressure on the vessel wall. 

The absolute shear stress varies throughout the cardiac cycle because of its pulsatile nature. In regions where stable flow is unidirectional with no recirculation, the time-averaged fluctuations in shear stress are positive (forward flow). Such laminar flow is known as mean positive shear stress. Temporal shear-stress gradients are defined as the increase or decrease of shear stress over a small period of time at the same location. Spatial shear-stress gradients are defined as the difference of shear stress between two close points of an endothelial cell at the same point in time. Mean positive shear stress greater than 6 dyne/cm^2^ predominates throughout much of the major systemic arterial vasculature. Recirculating flow occurs mainly around branch points and distal to areas of stenosis. The interaction between the hemodynamic forces generated by this flow and the systemic vascular endothelium may cause local formation of atherosclerotic lesions. It is important to note that the flow profiles within recirculation zones should not be confused with turbulent flow, since turbulence implies random movement of elements in the flow field. Turbulent flow accounts for a very small fraction of the total systemic flow. 

The morphology of endothelial cells within regions of recirculating flow is significantly different from cells located within regions of mean positive shear stress [17, 18]. Cells in these regions are not aligned and are characterized by a rounded shape, an increased proliferation rate and increased permeability [19-22]. Endothelial cells located within regions of mean positive shear stress are aligned with their longitudinal axis parallel to the direction of blood flow [17, 18]. This orientation effectively decreases drag resistance [23]. Based on these physiological observations it appears that mean positive shear stress acts as an endothelial cell survival factor rather than a growth factor [24]. 

Based on studies characterizing the force-transduction pathways [25-41]. White and Frangos [42] hypothesize that “hydrodynamic shear destabilizes the plasma membrane, leading to…… an increase in membrane free volume. Changes in membrane microviscosity directly activate various secondary signal cascades linked to heterotrimeric G protein. Tension generated across the cell membrane by fluid shear stress is transmitted to the cell-cell junction where known shear-sensitive proteins are localized. Furthermore, endothelial cell differentiation between mean positive shear stress and temporal gradients in shear stress takes place primarily in the cell-cell junction and is dictated by the rate of tension generated between the two flow profiles.”

### (2) Pulmonary vascular remodeling: effects of pathological pulmonary blood flow on pulmonary vascular structure.

Studies in animals and humans performed in the current era support the causal relationship between pathological pulmonary blood flow and the development of pulmonary hypertension [43-45]. The main mechanisms that may increase PVR in children with heart disease are left atrial hypertension due to systemic ventricular dysfunction, anatomic obstruction to pulmonary venous return, pulmonary veno-occlusive disease, pulmonary arteriolar constriction, anatomic obstruction of the large pulmonary arteries, increased pulmonary blood flow in CHD with left to right shunting, accessory sources of pulmonary blood flow from aortopulmonary collaterals, and sluggish pulmonary blood flow in children with single ventricle physiology following the Fontan procedure.

In animals, both increased [46], and decreased flow [47] may induce adverse responses in the pulmonary vasculature. The effects of both pathologically high and low shear, compared to physiological shear, on pulmonary endothelial production of vasodilating and vasoconstricting factors were investigated by Li and colleagues [48] using monolayers of bovine pulmonary arterial endothelial cells and pulmonary arterial smooth muscle cells exposed to varying shear conditions. They found that pathologically high and low flow attenuated endothelial release of nitric oxide and prostaglandin F_1a_, and enhanced release of endothelin-1. A mediator production profile that favors vasoconstriction. 

In patients with congestive heart failure (CHF), persistent elevation of left-ventricular end-diastolic pressure causes passive pulmonary venous congestion and reactive pulmonary vasoconstriction. At this preliminary stage, PVR is readily reversible with pulmonary vasodilators. However, persistent pulmonary venous congestion causes remodeling of the pulmonary arterial wall due to abnormalities of the elastic fibers, intimal fibrosis and medial hypertrophy. Pulmonary hypertension secondary to structural remodeling is referred as fixed because it is resistant to pharmacological treatments. Delgado and colleagues [49] studied the pulmonary vascular morphology of 17 adult HT recipients with preoperative CHF associated with ischemic heart disease, idiopathic dilated cardiomyopathy, valvular heart disease and constrictive pericarditis who died shortly after transplantation. Their main pathologic finding was medial hypertrophy of muscular pulmonary arteries. 

In an older era, Rabinovitch and colleagues [50] performed analysis of lung tissues obtained intra-operatively from 50 patients, mostly under 2 years of age (range 2 days-30 years) who underwent repair of ventricular septal defect, d-transposition of the great arteries, and atrioventricular canal. The findings were correlated with hemodynamic data obtained both pre and postoperatively. Three structural changes were observed and graded A, B, and C based on their severity (Table **1**). A similar study performed by the same group at the Children’s Medical Center, Boston (July 1976 to January 1981) [51] included performance of a lung biopsy at the time of the surgical repair on 74 patients with CHD who either had pulmonary hypertension or in whom it would be likely to develop if the lesion was not corrected. Structural changes were assessed based on the grading system described above and also according to the classification of Heath and Edwards [52] (Table **1**). Patients whose preoperative PVR was markedly elevated underwent biopsy first. If their pulmonary Heath-Edwards grade was IV to VI they were excluded from definitive repair. For the other patients, morphometric findings and Heath-Edwards grading were correlated with hemodynamic data from a cardiac catheterization performed immediately after surgery and about 1 year after surgery. Normal mean pulmonary pressure was taken as 18 mmHg or less and normal PVR index as less than 3.5 Wood units • m^2^. The Heath-Edwards grading system was predictive of pulmonary hypertension in the early but not late post-operative period. The study findings suggested, at least for patients undergoing an operation in the first 2 years of life, that even severe intimal change (Heath-Edwards grade III) is not prognostic of persistent elevation in PVR if growth and development of the pulmonary arteries has been relatively normal (morphometric grade A or B (mild)). Patients who underwent surgical correction in the first 8 months of life had normal pulmonary hemodynamics 1 year after repair despite marked structural abnormalities at the time of repair. Some of the patients operated on at ≥9 months of age that had pulmonary structural abnormalities as severe as in those operated on earlier had persistent elevation of PVR. Possible mechanisms are less complete regression of the abnormal findings, limited potential for growth of new normal vessels, or both. In patients with left to right shunt pulmonary vascular muscularization is accelerated, possibly because of mechanical or humoral growth stimulation generated by supra-physiological shear stress (see discussion of this term below) [53]. One possible cause of intimal hyperplasia in patients with CHD is endothelial over-regeneration in response to partial denudation of the endothelium of the more proximal intra-acinar arteries [54-56] .

## TREATMENT OPTIONS FOR PATIENTS WITH END-STAGE HEART FAILURE AND FIXED PVR

### Special Transplantation Techniques

Pulmonary hypertension is a risk factor for early and late mortality after heart transplantation. Gajarski and colleagues [57] studied the perioperative and intermediate outcomes in pediatric cardiac transplant recipients who had elevated PVR indexes preoperatively. They found that the vascular reactivity to pulmonary vasodilators and not the absolute PVR determined orthotopic HT suitability, and that post-transplant donor right heart failure is unlikely if pre-transplant recipient PVR index was ≤ 4 Wood units • m^2^ with vasodilator therapy. Studies in adults revealed that mortality after HT is increased if pharmacologic interventions are not able to reduce PVR below 2.5-3.5 Wood units • m^2^ [58, 59] . A PVR that cannot be reduced below this threshold with pulmonary vasodilators is usually termed fixed PVR. Possible treatment options for patients with end-stage heart failure and fixed PVR are as follows:

1. Heterotopic (“piggy back”) HT that allows preservation of the entire native heart. The latter provides assistance to the vulnerable transplanted right ventricle, however, the failing recipient left ventricle places the recipient at risk for lethal arrhythmias, systemic emboli, and compression of the left lung [60, 61].
2. Heart-lung transplantation. This procedure eliminates the diseased pulmonary vasculature, however, donors for heart-lung transplantation are rare, and the procedure has inherent long-term sequelae mainly related to its pulmonary component.
3. Right ventricle-sparing heart transplant was attempted in a canine model [62, 63]. The aim of this procedure is to preserve the recipient's right ventricle, which is already conditioned to pulmonary hypertension.
4. Transplantation using hearts from donors with idiopathic pulmonary hypertension for recipients with high PVR (domino procedure). This is a viable but rare option in transplant centers where patients undergo heart and lung transplantation for idiopathic pulmonary hypertension [64].

## HEMODYNAMIC UNLOADING

A recent laboratory study performed by O’Blenes and colleagues [65], tested the hypothesis that hemodynamic unloading leads to regression of the structural changes associated with obstructive pulmonary vascular disease. To do that they developed a model in which hypertensive rat lungs with experimental pulmonary vascular disease were hemodynamically unloaded by transplantation into syngeneic, normotensive recipient rats. The initial stage of the experiment included right middle and lower lobectomy and subsequent systemic injection of the toxin monocrotaline. With these interventions the investigators caused pulmonary vascular disease to the remaining right upper lobe and left lung by increased blood flow combined with monocrotaline toxic endothelial injury. The second stage was performed 28 days following these interventions and included left lung harvest and transplantation. That procedure caused hemodynamic unloading of the transplanted lung since pulmonary blood flow was probably directed preferentially to the normal native lung (low resistance circuit). The findings were as follows: [1] Pulmonary artery pressures were normal 14 days after transplantation and only mildly elevated by day 28. [2] Right ventricle hypertrophy did not develop in the recipient animals. [3] Medial hypertrophy and peripheral muscularization improved. [4] Pulmonary artery density, although somewhat improved, remained markedly below baseline levels.

Following that experiment, hemodynamic unloading of patients with fixed pulmonary hypertension with pulsatile and nonpulsatile ventricular assist devices led to reduction of PVR and allowed listing for orthotopic HT after a relatively short period of support (3-6 months), Furthermore, survival of these patients after transplantation was comparable with that of patients without prior pulmonary hypertension [66-77]. Based on our own experience [78], hemodynamic unloading with biventricular assist device combined with medical pulmonary vasodilator therapy leads to reversal of fixed pulmonary hypertension related to CHD. We have implanted Berlin Heart biventricular assist device (Berlin Heart AG, Berlin, Germany) in 13 patients from April 2005 to August 2008 [79] . The median age of the patients was 2 years (12 days to 17 years). The etiology of heart failure was cardiomyopathy in 11 children and CHD in 2. In those 2 patients with CHD, PVR index was greater than 10 Wood units • m^2^ unresponsive to pulmonary vasodilator therapy. PVR index decreased to 1.4 and 4.6 Wood units • m^2^ after 33 and 41 days of support, respectively. Both patients underwent orthotopic HT. Their PVR index remained normal without pulmonary vasodilator therapy within 3 months after transplantation.

## AT THE BEDSIDE: PRACTICAL CLINICAL APPROACH

### Two-ventricle Physiology

A child with end-stage HF adequately treated with oral anti CHF medications who presents with acute decompensated HF (ADHF) is admitted to the cardiac intensive care unit (CICU) for close non-invasive and invasive hemodynamic monitoring. The initial treatment line for these patients is inotropic support. A recent review of the pharmaceutical management of decompensated heart failure syndrome in children discusses in detail the indications, mechanism of actions, and dosages of the various available inotropes [80]. At St. Louis Children’s Hospital, the hemodynamically unstable child is started on dopamine, dobutamine or epinephrine as first line of inotropic treatment. If the child is stable hemodynamically, milrinone or dobutamine are initiated. Following an initial stabilization period, a right heart cardiac catheterization is performed to delineate the TPG and PVR and determine the child’s candidacy for HT. Assessment of the hemodynamic profile with a pulmonary artery (PA) catheter is an acceptable alternative in older children and allows titration of inotropic support to increase CO. 

### Treatment Options Based on Different Possible Hemodynamic Profiles

1. Elevated but reactive PVR in the context of elevated LA pressure. First line of treatment is inotropic support combined with IV diuretics. The second treatment line is intubation and positive ventilation. The latter is a potent afterload reducing method by way of decreasing the left ventricular (LV) transmural pressure and hence wall tension. Intubation of a child with ADHF may cause acute hemodynamic collapse and cardiac arrest because of acute reduction of sympathetic tone with the preintubation induction. The only indication to proceed is evidence of end-organ dysfunction and anaerobic metabolism. The third line of treatment, if systemic perfusion remains inadequate is mechanical circulatory support. Treatment with pulmonary vasodilators in this subset of patients prior to initiation of MCS may be risky since it may worsen pulmonary edema. The decompression of the LV by a VAD in these patients is usually sufficient to decrease PVR, again with no need to treat with pulmonary vasodilators.
2. Fixed PVR in the context of LA hypertension. Treatment of these patients is determined by their hemodynamic status and systemic perfusion. Treatment is targeted at optimization of CO. The combination of pulmonary hypertension and LV failure usually results in inadequate systemic perfusion and shock because of ventricular interdependence and underlying LV systolic and diastolic dysfunction. These patients usually require aggressive cardiopulmonary support comprised of positive pressure ventilation, inotropic support, sedation and neuromuscular blockade. It is our opinion that pulmonary vasodilators in this subset of patients are not beneficial. Reverse remodeling of the pulmonary vasculature requires lowering the LVEDP. Achieving that with inotropic support and mechanical ventilation is questionable and may not be sustainable. Other considerations are the debilitation and muscle degeneration that develops during prolonged mechanical ventilation and prolonged use of neuromuscular blockade. Based on our experience reverse remodeling of the pulmonary vasculature using mechanical circulatory support can be achieved in children with CHD, and therefore, in this particular group of patients, we recommend early VAD implantation.

## SINGLE-VENTRICLE PHYSIOLOGY

Children with single ventricle physiology may develop ADHF following each one of the palliation stages. The initial management of these patients is similar in principal to the management of children with two-ventricle physiology with ADHF, and includes inotropic support and IV diuretics. Mechanical ventilation in children with ADHF following the Glenn and Fontan procedures may be problematic since it may increase their PVR, however, this consideration is offset by the need to minimize work of breathing and oxygen consumption. Their TPG and PVR are assessed in the cardiac catheterization laboratory following a period of stabilization. While severe, fixed elevation of pulmonary vascular resistance that would preclude heart transplantation are unlikely in patients with cavopulmonary connections, nonpulsatile pulmonary flow can be associated with mild to moderate elevations of pulmonary vascular resistance that can result in primary right heart graft failure after transplant. [81] Inhaled nitric oxide and other pulmonary vasodilators may be beneficial in single ventricle patients with reactive pulmonary vascular bed [82, 83]. We have observed an association with aortopulmonary collaterals and elevation of pulmonary resistance in single ventricle heart transplant candidates [81] and an aggressive strategy to identify and embolize these collaterals prior to transplant may be beneficial. 

Experience with MCS in these patients other than ECMO is very limited. The literature reveals only sporadic case reports describing long-term MCS in the context of single-ventricle physiology [84-86]. With the current availability of the Berlin Heart, it is likely experience with support of single ventricle physiology in infancy, after the bidirectional Glenn shunt, and in the failing Fontan patient will accumulate rapidly to allow for an evaluation the efficacy of VAD therapy to decrease elevated pulmonary resistance in this unique patient population.

## SPECIAL CONSIDERATIONS

Patients with CHD associated with unequal distribution of pulmonary blood flow may develop pulmonary vascular remodeling in one lung whereas the second lung may have normal pulmonary vasculature or only minimal structural changes leading to PVR elevation amenable to medical treatment. Unequal pulmonary blood flow distribution can be found in patients with Tetralogy of Fallot and pulmonary atresia with major aortopulmonary collaterals. Another possible cause for pulmonary blood flow maldistribution is partial pulmonary venous obstruction involving pulmonary venous egress of one lung. Heart transplantation has been successfully performed in such patients [87]. Pre-transplantation embolization of aortopulmonary collaterals, or ligation of these collaterals during transplantation may decrease the risk of primary graft failure due to volume overload [88, 89]. Another subset of patients that deserve special attention is that of patients with hypoplastic left heart syndrome and intact atrial septum. In spite of minimal pulmonary blood flow in utero, this combination is associated with nonimmune fetal hydrops [90] and congenital pulmonary cystic lymphangiectasis [91] and may result in prenatal mortality. Of those surviving to term, an immediate atrial septostomy is required after delivery because of severe hypoxemia. Even if no significant end organ damage develops in the immediate postnatal period, the outcome of these infants is still guarded due to maldevelopment of the pulmonary vasculature. Rychik and colleagues [92] identified three types of intact atrial septum by echocardiography: 1) type A is consistent of large left atrium, thick prominent septum secundum with thin septum primum adherent to each other; 2) type B is consistent of a small left atrium with thick, muscular atrial septum, and 3) type C that consists of a giant left atrium, thin atrial septum with severe mitral regurgitation. Lung tissue specimens were obtained from six patients. Most striking findings were in the type B patients. The lymphatics were severely dilated and the pulmonary veins were thick, dilated and “arterialized” with multiple elastic laminae noted. The pulmonary vascular pathology, especially in type B patients may preclude these patients from the single ventricle palliation pathway. The question is if orthotopic heart transplantation alone is sufficient or whether heart-lung transplantation is required. There is no clear-cut answer since outcome data are minimal. Preliminary investigations should include: 1) a cardiac catheterization to assess the degree of pulmonary venous obstruction and PVR as well as the reactivity of the pulmonary vascular bed to pulmonary vasodilators; 2) an open lung biopsy. Early listing for either heart transplantation or heart-lung transplantation in patients that are not amenable for the Norwood procedure may be helpful in accruing time on the list since waiting time may be very long. 

## CONCLUSION

In patients with CHD and end-stage heart failure, pulmonary remodeling caused by abnormal pulmonary blood flow profiles may result in severe pulmonary hypertension refractory to pulmonary vasodilators, also known as fixed pulmonary hypertension. This entity is considered a contraindication to orthotopic heart transplantation [93]. This entity is encountered more frequently in transplant candidates with congenital heart disease. Even in congenital heart disease patients who show reversibility of pulmonary resistance and evidence of “acceptable” hemodynamics, the uncertainties often involved in the measurement of pulmonary resistance in congenital heart disease candidates may often lead to primary graft failure from right heart failure after transplant. Careful planning of heart transplant procedures in this subgroup of patients in preparation for this complication is warranted as are ongoing efforts to minimize pulmonary resistance in these patients prior to transplant.

Until recently, the treatment of patients with heart failure and fixed PVR was limited almost exclusively to combined heart-lung transplantation. However, over the course of the last decade, the concept of reverse remodeling of the pulmonary vasculature with hemodynamic unloading of the heart has been confirmed both in adults and children supported with ventricular assist devices. This option opens new treatment options for these patients. The definition of “fixed” pulmonary hypertension refractory to pulmonary vasodilator treatment that would preclude heart transplant in the current era of pediatric VAD availability is evolving as these new technologies are applied to congenital heart disease patients. 

## Figures and Tables

**Table 1:** Grading of Pulmonary Vascular Pathology in Patients with CHD and Excessive Pulmonary Blood Flow.

Morphometric Grade	Heath-Edwards Grade	Morphometric Findings	Heath-Edwards Histopathological Findings	Pulmonary Hemodynamic Profile
A	N	Extension of muscle into peripheral arteries normally nonmuscular, either as a solitary finding or associated with a mild increase in the medial wall thickness of the normally muscular arteries (≤1.5 normal).	no striking evidence of medial hypertrophy, same as in the morphometric grade A	Increased pulmonary blood flow without evidence of increased pulmonary artery pressure.
B		Grade A findings with greater medial hypertrophy		
B (mild)	N	medial wall thickness is greater than 1.5 but less than 2 times normal	no striking evidence of medial hypertrophy, same as in the morphometric grade B(mild)	
B (severe)	I	wall thickness is ≥2 times normal.	medial hypertrophy can be appreciated subjectively as in morphometric grade B (severe)	associated with pulmonary arterial hypertension.
C		Grade B (severe) findings with a reduced number of peripheral arteries relative to alveoli and usually decreased arterial size		Moderate-to-severe elevation in pulmonary vascular resistance
C (mild)		more than half the normal number of arteries is present		
C (severe)		when half the normal number of arteries or less is present.		
	II		presence of eccentric or concentric intimal hyperplasia	
	III		occlusive intimal hyperplasia with hyalinization of the media	
